# Psychological issues in breast cancer survivors confronted with motherhood: Literature review and a call to action

**DOI:** 10.3389/fpsyg.2023.1133204

**Published:** 2023-03-02

**Authors:** Valentina Elisabetta Di Mattei, Gaia Perego, Paola Taranto, Martina Mazzetti, Federica Ferrari, Noemi Derna, Fedro Alessandro Peccatori, Giorgia Mangili, Massimo Candiani

**Affiliations:** ^1^School of Psychology, Vita-Salute San Raffaele University, Milan, Italy; ^2^Clinical and Health Psychology Unit, IRCCS San Raffaele Scientific Institute, Milan, Italy; ^3^Centro InTerapia, Monza, Italy; ^4^Fertility and Procreation Unit, Division of Gynecologic Oncology, Department of Gynecology, European Institute of Oncology IRCCS, Milan, Italy; ^5^Obstetrics and Gynecology Unit, IRCCS San Raffaele Scientific Institute, Milan, Italy; ^6^School of Medicine, Vita-Salute San Raffaele University, Milan, Italy

**Keywords:** breast cancer, motherhood, pregnancy, psychological symptoms, breastfeeding

## Abstract

Breast cancer is currently the most common cancer among women worldwide; in 15–25% of cases, patients are premenopausal at the time of diagnosis, and 50% of women desire pregnancy after cancer diagnosis. Motherhood after breast cancer involves complex psychological challenges with long-term consequences, though it is safely pursuable with adequate support. The purpose of this mini-review is to analyze the psychological implications surrounding pregnancy and motherhood after breast cancer and promote action in addressing the challenges that might affect women facing these life events.

## Introduction

Breast cancer is currently the most common cancer among women worldwide, with 2.26 million cases recorded in 2020 (WHO, 2022). In 15–25% of cases, patients are premenopausal at the time of diagnosis (De Pedro et al., 2015), in fact approximately 7 to 10% of women diagnosed with breast cancer are younger than 40 years old (Rossi et al., 2019). For women who survive cancer, fertility and reproductive problems are of the utmost importance and almost 50% of young women desire pregnancy after breast cancer diagnosis (Paluch-Shimon et al., 2017).

However, a meta-analysis shows that the pregnancy rate after breast cancer treatment was on average 40% lower than the general population pregnancy rate (Gerstl et al., 2018).

This reflects both the damage to fertility caused by cancer treatments and the concerns of caregivers and patients about a possible negative impact of pregnancy on a woman’s prognosis, although there is no evidence for the latter in the literature (Hartman and Eslick, 2016; Condorelli et al., 2021).

The aim of this work is to review the available literature on psychological issues in breast cancer survivors facing motherhood to better manage the unmet needs of this group of patients.

### Mood status and health concerns

Accepting cancer diagnosis, undergoing treatments, managing possible side effects, and facing an uncertain future are steps in a stressful process that can result in psychological instability and depression (Dinapoli et al., 2021).

A large percentage of breast cancer patients experience multiple concomitant psychological symptoms during the cancer treatment journey, such as distress, anxiety, cognitive impairment, and sexual dysfunction (Guimond et al., 2019).

A recent study shows that women who develop reproductive problems after anticancer treatment experience more depressive symptoms over time (Nejatisafa et al., 2020).

The dilemma regarding childbirth that young cancer survivors face is not just of a medical nature, it is also influenced by psychological factors.

Pregnancy after breast cancer comes with unique and complex psychological and relational challenges with potential long-term consequences for patients and their families. Women who have had an oncological diagnosis in the past often face uncertainty about the outcome of the disease or the discontinuation of hormone therapy, which causes ambivalent feelings about pregnancy (Alder and Bitzer, 2008; Ives et al., 2016). In fact, the most common experiences include returning to normalcy and recovering from illness, but also being concerned for the health of their children, fearing relapses, and not seeing their children grow up (Gorman et al., 2010; Kuswanto et al., 2018; Faccio et al., 2020). The systematic review of Schmidt et al. (2016) aligns with these results, revealing how pregnant women with a history of breast cancer present gestation concerns to a greater extent than women with other cancer diagnoses. These concerns were primarily associated with fear of recurrence, fear of tumor progression due to pregnancy, and misinterpreting breast changes that they may experience with pregnancy (Schmidt et al., 2016).

In the literature, some studies have demonstrated that negative emotional states elicited by the disease, such as anxiety, anguish, anger, and fatigue, increase during pregnancy (Alder and Bitzer, 2008; Henry et al., 2012; Faccio et al., 2020; Schwab et al., 2021). In contrast, the study by Mascheroni et al. (2019) found that mothers’ mood state during the last trimester of pregnancy did not differ between cancer survivors and women in the control sample. However, the same study also found that these women have significantly higher levels of post-traumatic stress disorder (PTSD) symptoms and lower quality of life levels. This evidence suggests that pregnancy after breast cancer represents a moment of vulnerability deserving special attention to prevent negative consequences on parenting (Gorman et al., 2010; Kuswanto et al., 2018).

Cancer survivors are also concerned about potential health problems for their children. One of the most common concerns is that past cancer treatment could lead to having a child with a birth defect or a genetic abnormality (Schover, 2005; Ghaemi et al., 2019).

The advent of genetic testing for hereditary cancer syndromes creates a new set of dilemmas for those who want to become parents. Carrying a deleterious BRCA mutation is associated with an increased lifetime risk of breast and ovarian cancer, so it is often recommended to get pregnant at a young age, followed by risk-reducing salpingo-oophorectomy. Many BRCA-mutated women are confronted with a true reproductive decision-making dilemma as a consequence of the 50% risk of transmitting the mutated gene to their children. Technologies such as prenatal diagnosis and preimplantation genetic diagnosis, in case of conception through *in vitro* fertilization or intracytoplasmic sperm injection, identify autosomal dominant mutations known to be responsible for hereditary cancer syndromes (Peccatori et al., 2018). The advantage of the preimplantation genetic diagnosis is that only unaffected embryos are transferred, thus avoiding a pregnancy termination if the fetus carries the undesirable genetic mutation. However, only few couples use this test for inherited cancer (Mor et al., 2018; Khouri et al., 2019).

### Pregnancy representation

Studies indicate that most cancer survivors have reproductive intentions 3–7 years after diagnosis and that the desire to have children is mainly associated with the desire for parenting before cancer treatment, age, and parity (Armuand et al., 2014). When these intentions are unsatisfied, high levels of emotional suffering are detected, with important consequences for psychological and social well-being (Canada and Schover, 2012; Bártolo et al., 2021). Studies evaluating the effectiveness of fertility-related interventions on patients’ mental health have shown that pre-treatment fertility counseling has improved patients’ physical and psychosocial quality of life (Meneses and Holland, 2014; Sigismondi et al., 2015; Ter Welle-Butalid et al., 2019).

Several aspects influence the importance that women give to motherhood after breast cancer; one example is having children before the diagnosis. In fact, the priority for these mothers is to protect the children they already have, and a new pregnancy could be considered dangerous for recurrence (Ives, 2009; Hartman and Eslick, 2016). However, this does not mean that women with children before the diagnosis do not wish to achieve a pregnancy after cancer. Indeed, pregnancy after a breast cancer diagnosis seems to represent rebirth, hope, and revenge on life (Ives et al., 2016; Ferrari et al., 2018). Creating or expanding their family means rebuilding a positive dimension with their partner and seizing their chances of redemption from the disease (Crawshaw and Sloper, 2010; Young et al., 2019). A recent study by Hsieh et al. (2018) illustrates a theoretical model according to which cancer and its treatment are considered elements that interrupt a woman’s normal life and destroy its balance. Every woman tries to find new meanings in her life during and after cancer; a child could represent the restoration of the original balance.

Another aspect that has only recently been considered is mother–child prenatal interaction (Ferrari et al., 2017; Faccio et al., 2020). Cancer survivors seem to show lower levels of interaction and affiliation with their fetus compared to women without a cancer diagnosis in their life history. Prenatal interaction is fundamental for the future mother–child relationship in the postpartum period and for the development of the child’s personality (Graignic-Philippe et al., 2014; Ferrari et al., 2018; Faccio et al., 2020).

### Delivery and preparation

Childbirth is considered a stressful life event; between 1 and 6% of women in the first year of birth can suffer from PTSD following a difficult birth experience. In addition to the diagnosed cases, about 30% of women evaluate their experience as traumatic (Soet et al., 2003).

The presence of psychiatric problems before birth and traumatic life events has been associated with symptoms of PTSD after birth in numerous studies (Kennedy and MacDonald, 2002; Cohen et al., 2004; Cigoli et al., 2006; O’Donovan et al., 2014). A cancer diagnosis is considered by the literature as a traumatic event and for this reason a potential risk factor for the development of psychological problems following a complicated birth (Mehnert and Koch, 2007).

The effects of anxiety and stressful life events on women’s health during pregnancy and their birth outcomes have been studied. Stress and anxiety may be associated with numerous adverse outcomes such as preeclampsia, prolonged labor, preterm labor, and delivering a low-birth-weight infant (Graignic-Philippe et al., 2014). A negative birth experience could adversely affect postpartum maternal mood, the mother–child bond and breastfeeding (Weisman et al., 2010; Moloney and Gair, 2015; Bell et al., 2019).

Childbirth is a multidimensional life event, and women simultaneously report both negative and positive aspects of childbirth. These include pain, anxiety, and a loss of control, as well as a sense of accomplishment and joy or satisfaction (Waldenström et al., 1996; Van Teijlingen et al., 2003; Hoffmann and Banse, 2021).

Considering the possible consequences of anxiety and distress on childbirth and of negative childbirth experience, a psychological intervention focused on the preparation and assistance to childbirth is recommended. Women should be encouraged to discuss their goals, expectations, and plans for birth. The psychologist can help women cope with difficulties during pregnancy by providing them with information about emotional reactions that are normal or expected and that may signal the need for specific interventions. Finally, women should be encouraged to process their birth experiences shortly after they occur. This opportunity may help them reconcile ambivalent feelings about the childbirth experience (Howarth et al., 2010).

### Marital and family support

The decision to have a child is a shared decision for the couple and the support of the partner in determining positive psychological outcomes during the gestational period is extremely important (Webster et al., 2011; Stapleton et al., 2012; Ives et al., 2016). Studies suggest that a supportive partner can be a protective factor against depression during pregnancy and postpartum, with benefits also for the well-being of the baby (Stapleton et al., 2012). Anxiety and depression levels during pregnancy are higher in women with poor family support (Cheng et al., 2016). Moreover, poor social support from friends, family and partners is associated with a woman’s lower quality of life (Webster et al., 2011; Lagadec et al., 2018).

The comparison between women without an oncological history and those who have previously had a breast cancer diagnosis highlights that the latter perceive greater support from their partners and consider them figures who can assume a protective role toward them. The support given by the partner in the evaluation of fertility conservation options at the time of diagnosis and the choice of the method and timing of conception after treatment is also relevant (Faccio et al., 2020).

These results provide the first evidence of the importance of social support for the quality of maternal life during gestation and in the first months after childbirth.

Counseling and interventions to reduce depression and improve quality of life after childbirth should focus on the mother’s social support network. Ideally, these interventions should be undertaken during pregnancy following a careful evaluation of the woman’s support system (Webster et al., 2011; Lagadec et al., 2018).

According to the literature and clinical experience, psychological counseling should also focus on the exploration of ambivalent and negative emotions that can develop regarding pregnancy and which can be normal in these circumstances. The evaluation and empowerment of coping strategies can help women cope with difficult situations (Florsheim et al., 2012; Kaboli et al., 2017).

Support from a mental health professional can be an important resource because, although relevant, social and family support can also have a negative impact. Family members are often emotionally close to the patient and will have their fears and worries, which might make their support less effective (Ives et al., 2016).

### Breastfeeding

Scientific evidence shows that breast milk is the ideal food for both the infant and the mother because of the physical and psychological benefits associated with it (Martin et al., 2016). The use of artificial nutrition and the early cessation of breastfeeding seem to increase the risk of developing certain diseases such as obesity, gastroenteritis, otitis, respiratory infections, and type 1 and type 2 diabetes in children (Bartick et al., 2009; Gianni et al., 2019). From a psychological point of view, breastfeeding is associated with slightly better performance in cognitive development tests than those obtained by artificially breastfed babies (Gartner et al., 2005; Lopez et al., 2021).

Regarding the health of the mother, studies on the benefits of breastfeeding report: a reduction in postpartum bleeding and a faster uterine involution due to the increased concentration of oxytocin; an earlier return to the weight women had before pregnancy (Gartner et al., 2005); and a reduction in the risk of developing breast or uterus cancer and type 2 diabetes (Bartick et al., 2009; Jelly and Choudhary, 2019). A meta-analysis with 27 studies involving 13,907 breast cancer cases suggested that breastfeeding was inversely associated with the risk of breast cancer (Zhou et al., 2015).

Concerning the benefits for psychological health, natural breastfeeding promotes a greater state of general relaxation, positive emotionality, and satisfaction with the care of the baby: women who breastfeed naturally turn out to be calmer, less anxious and stressed than those who feed artificially (Groër, 2005; Krol and Grossmann, 2018).

Breastfeeding also seems to have a fundamental neuro-biological role in the formation of a good mother-baby bond: high concentrations of prolactin and oxytocin are related to a better quality of maternal behavior and post-natal attachment (Levine et al., 2007; Walter et al., 2021).

Currently, there are no reliable epidemiological data regarding breastfeeding after breast cancer. The Society of Obstetricians and Gynecologists of Canada (SOGC) guidelines indicate that women previously treated for breast cancer should be encouraged to breastfeed, as there is no evidence that this practice is risky for the health of the mother or the baby. However, a recent systematic review by Bhurosy et al. (2021) shows that breastfeeding might be challenging among breast cancer survivors. According to the authors, although breastfeeding is possible and the treated breast is able to produce milk, many breast cancer survivors experience other significant challenges such as uncertainty about breastfeeding, lack of support from physicians and family members, lack of access to an International Board-Certified Lactation Consultant (IBCLC), and nipple pain and discomfort. Nonetheless, there are several clinical and social factors associated with safe and possible breastfeeding. Social factors include being motivated to breastfeed and receiving counseling and support from a multidisciplinary team of health professionals, family members or friends. Clinical factors include the use of the contralateral breast, lactation counseling and advice from an IBCLC, frequent feedings and use of galactagogues (Bhurosy et al., 2021).

Studies on healthy women have shown that maternal psychological variables influence the choice of breastfeeding and its duration (O’Brien et al., 2008; De Jager et al., 2014; Dagla et al., 2021). In particular, it has been shown that anxiety, neuroticism as a personality trait and a body image disorder negatively affect the intention to breastfeed: the more they are present, the less the mother is willing to breastfeed (Roth, 2006; Di Mattei et al., 2016).

Body image describes the cognitive, affective, and behavioral aspects of one’s body (Cash et al., 2002; Hosseini and Padhy, 2021). Negative body image is common in women who have been diagnosed with breast cancer, particularly as the breast is a symbol of femininity and sexuality in western society (Kolodziejczyk and Pawłowski, 2019). For these women, the breast becomes a potentially lethal sick organ, and for the surviving women who are experiencing pregnancy it may be difficult to re-accept that sick breast as a life-giving and nourishing organ for their baby (Hopwood, 1993; Brown et al., 2015).

The literature has shown that low self-esteem, guilt, and stigma are present in women who are unable to breastfeed (Bresnahan et al., 2020). The difficulties that women with previous breast cancer encounter in breastfeeding can cause a sense of inadequacy compared to their role as mothers because they fear that this will prevent them from building a positive relationship with their baby (Alder and Bitzer, 2008; DiPietro, 2010).

In a qualitative analysis conducted by Gorman et al. (2009), women’s fears about breastfeeding after breast cancer emerged significantly. These fears included uncertainties about the possibility of breastfeeding, fear of breastfeeding with one breast and fear of not having enough milk.

Maternal counseling in pregnancy and breastfeeding is essential to prevent negative effects on the mental health of women and children and their bond. The reduced production of milk by the previously affected and irradiated breast, the adequacy of the quantity and quality of the milk are issues that must be addressed with competence and patience (Azim et al., 2009; Bhurosy et al., 2021).

Denying breast cancer survivors the opportunity to breastfeed remains unjustified in the absence of supporting evidence. Addressing this issue would help increase the perception of a return to normalcy and improve the quality of life of these women (Azim et al., 2010; Bhurosy et al., 2021).

## Conclusion

The most recent trends in breast cancer epidemiology show that incidence among women aged 20–49 years is gradually increasing (Ellington et al., 2022); at the same time there is evidence that the age at first pregnancy is increasing as well (Eurostat, 2021).

This calls for action in addressing the issues that might affect women seeking and facing pregnancy after breast cancer diagnosis and treatment, which both represent challenging events for a woman. For this reason, a multidisciplinary approach is recommended since diagnosis, providing women with all the necessary tools to preserve their fertility before starting cancer treatment (Di Mattei et al., 2020, 2021) and providing adequate support throughout the pregnancy and breastfeeding journey afterwards. Receiving adequate information about fertility and the possibility of a safe pregnancy after breast cancer, while feeling supported by a multidisciplinary team including the psychologist, might promote a better adjustment to the disease and increase psychological well-being and quality of life in the long-term. Finally, involving patients’ partners is crucial to promote communication and a shared path within the couple.

## Author contributions

VEDM, GP, GM, FF, FAP, and MC contributed to conception and design of the manuscript. FF and GP wrote the first draft of the manuscript. PT, MM, ND, and VEDM wrote sections of the manuscript. All authors contributed to manuscript revision, read, and approved the submitted version.

## Conflict of interest

The authors declare that the research was conducted in the absence of any commercial or financial relationships that could be construed as a potential conflict of interest.

## Publisher’s note

All claims expressed in this article are solely those of the authors and do not necessarily represent those of their affiliated organizations, or those of the publisher, the editors and the reviewers. Any product that may be evaluated in this article, or claim that may be made by its manufacturer, is not guaranteed or endorsed by the publisher.
